# Management of choroid plexus tumors—an institutional experience

**DOI:** 10.1007/s00701-019-03832-5

**Published:** 2019-02-19

**Authors:** Arthur Hosmann, Felix Hinker, Christian Dorfer, Irene Slavc, Christine Haberler, Karin Dieckmann, Engelbert Knosp, Thomas Czech

**Affiliations:** 1 Department of Neurosurgery, Medical University of Vienna, Waehringer Guertel 18–20, 1090 Vienna, Austria; 2 Comprehensive Cancer Center–Central Nervous System Tumors Unit (CCC-CNS), Medical University of Vienna, Vienna, Austria; 3 Department of Pediatrics and Adolescence Medicine, Medical University of Vienna, Vienna, Austria; 4 Institute of Neurology, Medical University of Vienna, Vienna, Austria; 5 Department of Radiotherapy, Medical University of Vienna, Vienna, Austria

**Keywords:** Atypical choroid plexus papilloma, Choroid plexus carcinoma, Choroid plexus papilloma, Choroid plexus tumor, Gross-total resection, Infiltration

## Abstract

**Background:**

Choroid plexus tumors are rare entities. Resection is the mainstay of treatment in grade I and grade II tumors and adjuvant treatment is usually reserved for the less frequent choroid plexus carcinoma (CPC). Outcome is not only related to their histological grade but also dependent on their size, location, and presence of often multifactorial disturbances of cerebrospinal fluid (CSF) circulation.

**Methods:**

Retrospective analysis of 36 consecutive patients operated on a choroid plexus tumor at our institution in a mixed pediatric and adult population between 1991 and 2016.

**Results:**

Twenty-one CPP, 11 atypical choroid plexus papillomas (aCPP), and four CPC were encountered in 17 children and 19 adults. Regardless of histological grading, gross-total resection (GTR) could be achieved in 91.7% of patients. Tumor recurrence (25.0%) was significantly associated with histological grading (*p* = 0.004), subtotal resection (*p* = 0.002), and intraoperatively evident zones of tumor infiltration (*p* = 0.001). Adjuvant therapy was performed in 19.4% of patients, mainly diagnosed with CPC. The 5-year overall survival rate was 95.2% for CPP and 100.0% for both aCPP and CPC. Survival was related to the extent of resection (*p* = 0.001), tumor progression (*p* = 0.04), and the presence of leptomeningeal metastases (*p* = 0.002). Even after resection, either ventricular or subdural shunting was required in 25.0% of patients.

**Conclusions:**

We could confirm that GTR is crucial for treatment of choroid plexus tumors. Parenchymal tumor infiltration as detected intraoperatively was associated with the extent of resection and not limited to CPC. CSF disturbances mandating treatment may persist after resection.

## Introduction

Tumors of the choroid plexus epithelium are rare intraventricular lesions and account for 0.5% of brain tumors in both children and adults. In children, 10 to 20% are diagnosed within the first year of life [9, 20, 47, 49] and are usually located supratentorially. In adults choroid plexus tumors (CPT) are almost exclusively located infratentorially, representing a reverse situation compared to other tumor entities [15, 17, 47].

The World Health Organization (WHO) classification differentiates between benign choroid plexus papillomas (CPP; WHO I), aggressive malignant choroid plexus carcinomas (CPC; WHO III), and atypical choroid plexus papillomas (aCPP; WHO II) as an intermediate form. CPC occur mostly in very young children, are typically aggressive, and disseminated at diagnosis in more than 20% of cases [49].

Although CPTs occur in both children and adults, most case series focus exclusively on an either pediatric or adult population [7, 15, 17, 22, 24, 25, 30, 34, 35, 39, 50]. However, adequate treatment requires age-specific consideration for optimized outcome. Most patients with CPT can be cured by total resection alone, but perioperative morbidity due to the young age, the intraventricular location, and high tumor vascularization can also negatively affect patients’ outcome independent of histological grading [7, 13, 30, 42, 48]. Especially, the high rate of temporary CSF drainage and permanent shunting may contribute to an increased tumor-independent morbidity in these patients [22, 25, 30, 35].

Therefore, we present our 25-year experience in the surgical and postoperative management of CPT in a mixed pediatric and adult patient population. We put a particular focus on age-related tumor characteristics and on surgical considerations specifically with regard to cerebrospinal fluid disturbances, which are usually neglected in existing literature.

## Methods

### Patient selection

All consecutive patients operated on a histologically proven CPT at the Neurosurgical Department of the Medical University Vienna between 1991 and 2016 were included. All tissue samples were reviewed by the university’s neuropathology department and re-classified according to the WHO criteria 2016 for CPT. The study protocol was approved by the local ethics committee (EK 2005/2015).

### Patient characteristics

Details for individual cases are shown in Table 1.

**Table 1:** Demographics, clinics, resection details, and outcome of all included patients. CPA, cerebellopontine angle; CR, complete remission; DOD, dead of disease; f, female; GTR, gross-total resection; lat., lateral; LOF, lost of follow-up; m, male; SD, stable disease; SDP, subduro-peritoneal; STR, subtotal resection; VA, ventriculo-atrial; VP, ventriculo-peritoneal

	Patient no.	Age (years)	Sex	WHO	Localization	Metastasis	Resection	Infiltration zones	Adjuvant treatment	Relapse	No. of local tumor resections	Permanent CSF diversion	Follow-up (years)	Tumor state
Children	1	0.8	w	I	lat. ventricle	No	GTR	No	No	No	1	No	7.8	CR
	2	0.8	m	I	3rd ventricle	No	GTR	No	No	No	1	No	21.5	CR
	3	0.8	m	I	lat. ventricle	No	GTR	No	No	No	1	No	19.2	CR
	4	1.5	w	I	lat. ventricle	No	GTR	No	No	No	1	No	9.8	CR
	5	3.1	m	I	3rd ventricle	No	GTR	No	No	No	1	No	22.7	CR
	6	4.1	w	I	lat. ventricle	No	GTR	No	No	No	1	No	2.5	CR
	7	9.1	w	I	4th ventricle	No	GTR	Yes	No	No	1	No	13.6	CR
	8	10.2	w	I	lat. ventricle	No	GTR	No	No	No	1	No	22.6	CR
	9	0.2	w	II	lat. ventricle	No	GTR	No	No	Yes	2	SDP	6.7	SD
	10	0.3	w	II	lat. ventricle	No	GTR	Yes	No	No	1	No	15.2	CR
	11	0.6	m	II	lat. ventricle	No	GTR	No	No	No	1	No	0.3	CR
	12	1	w	II	3rd ventricle	No	GTR	No	No	No	1	No	0.3	CR
	13	1.2	m	II	3rd ventricle	No	GTR	No	No	No	1	SDP	3.1	CR
	14	0.5	m	III	lat. ventricle	No	GTR	Yes	Chemo	No	1	VP	1.3	SD
	15	2.8	m	III	lat. ventricle	At diagnosis	GTR	Yes	Chemo-radiation	No	1	VP, SDP	8.6	SD
	16	5.3	m	III	lat. ventricle	At diagnosis	STR	Yes	Chemo-radiation	Yes	2	VP	8.2	DOD
	17	6.5	w	III	lat. ventricle	No	GTR	Yes	Chemo	Yes	1	No	23.3	SD
Adults	18	22.7	w	I	CPA	No	GTR	No	No	No	1	No	16.8	CR
	19	27.2	w	I	4th ventricle	No	GTR	No	No	No	1	No	1.5	CR
	20	29	w	I	4th ventricle	No	GTR	No	No	No	1	No	8.7	CR
	21	31.3	w	I	4th ventricle	No	GTR	No	No	No	1	VA	17.4	CR
	22	40.2	w	I	CPA	No	GTR	No	No	No	1	No	0.1	LOF
	23	46.6	w	I	4th ventricle	No	GTR	No	No	No	1	No	0.1	CR
	24	48.5	w	I	4th ventricle	No	GTR	No	No	No	1	No	1.9	CR
	25	52	w	I	4th ventricle	No	GTR	No	No	No	1	VP	2.1	CR
	26	52.7	m	I	4th ventricle	No	GTR	Yes	No	Yes	1	No	20.6	SD
	27	53.2	m	I	CPA	No	GTR	No	No	No	1	No	0.0	DOD
	28	54.9	w	I	4th ventricle	No	GTR	No	No	No	1	No	0.0	LOF
	29	58.9	w	I	4th ventricle	No	GTR	No	No	No	1	No	18.5	CR
	30	60.9	w	I	CPA	No	GTR	No	No	No	1	No	0.3	LOF
	31	33.7	w	II	4th ventricle	Follow-up	STR	Yes	No	Yes	2	No	9.5	DOD
	32	34.7	m	II	4th ventricle	No	GTR	Yes	No	Yes	3	VP	15.8	SD
	33	46.8	w	II	4th ventricle	No	STR	Yes	Chemo	Yes	2	No	9.9	SD
	34	54	m	II	4th ventricle	No	GTR	Yes	Radiation	Yes	1	No	4.1	SD
	35	57.8	m	II	4th ventricle	No	GTR	Yes	Radiation	No	1	No	0.2	LOF
	36	70.9	m	II	4th ventricle	No	GTR	No	No	Yes	3	VA	9.0	DOD

Thirty-six consecutive patients were identified, including 17 children (median, 1.2 years; range, 0.2–10.2 years) and 19 adults (median, 48.5 years; range, 22.7–70.9 years). Sex distribution was nearly equal in children (male:female = 8:9). For adults, 68.4% of patients were female. In total, 21 CPP, 11 aCPP, and 4 CPC were present in our cohort.

Clinical symptoms at presentation are shown in Table 2, tumor localization and the surgical approach in Table 3. The median tumor size was 3.5 cm (range, 1.1–10 cm).

**Table 2:** Clinical presentation

	Children	Adults
Increased ICP	76.5%	15.8%*
Increased head circumference	41.2%	N/A
Headache	23.5%	68.4%*
Gait disturbance	5.6%	52.6%*
Vertigo	7.1%	36.8%*
Diplopia	11.8%	31.6%
Behavior	35.3%	5.3%*
Paresis	17.6%	10.5%
Incidential finding	5.9%	21.1%

*Significant differences between age groups (*p* < 0.05)

**Table 3:** Tumor localization and surgical approach

	Lat. ventricle	3rd ventricle	4th ventricle	CPA
Suboccipital	–	–	16	1
Transcortical	8	1	–	–
Transcallosal	4	2	–	–
Supracerebellar	–	1	–	–
Retromastoidal	–	–	–	3
Total	12	4	16	4

Dissemination as evidenced by MRI at diagnosis was present in two children with CPC, who presented both with spinal metastases.

### Clinical evaluation

In a retrospective chart review, patient demographics, tumor location, and postoperative characteristics were analyzed. Resection was categorized as either gross-total resection (GTR) or subtotal resection (STR) if residual tumor (< 10%) was found on postoperative contrast-enhanced MRI scans. Operative reports were reviewed for surgical details, focusing on evidence for tissue infiltration. Data for intraoperative blood transfusion management was based on anesthesiologic protocols. The decision for adjuvant chemotherapy and/or radiation was made on a case by case basis. Tumor progression was defined as tumor recurrence or progression of residual disease within the previous surgical field or detection of metastases on follow-up images. Overall survival (OS) was obtained from the Austrian death register in December 2016.

### Statistical analysis

Statistical analysis was performed using SPSS® Statistics 22 (IBM Corp., Armonk, NY). Contingency tables were evaluated using Fisher’s exact test. Group comparison was performed using the Mann-Whitney *U* test. Progression-free survival (PFS) and OS was estimated with Kaplan–Meier analysis. Predictors for PFS or OS were identified using the log rank test. Differences were considered to be statistically significant at a two-sided *p* value of < 0.05.

## Results

### Tumor resection

Parenchymal infiltration was observed in 32.2% of patients and significantly associated with histological grading (4/4 CPC, 6/11 aCPP, 2/21 CPP; *p* = 0.001).

GTR was achieved in 33 (91.7%) patients. In all three cases with subtotal resection (2 aCPP, 1 CPC), infiltration to medulla oblongata, thalamus, or caudal cranial nerves was present.

In one patient with disseminated CPC (patient no. 15), a solitary spinal metastasis was resected 10 days after first surgery.

### Intraoperative blood transfusion management

Complete anesthesia protocols were available for 22 patients (61.1%). Intraoperative blood transfusion was necessary for 5 out of 8 children with complete data (age, 0.5–5.3 years), including two CPC, one aCPP, and two CPP. The median volume of blood transfusion was 510 ml (range, 150–1100 ml amounting to 20–72% of estimated blood volume in these children). Tumor size was not significantly different in patients with blood transfusion (median size, 51.0 mm) in comparison to patients without transfusion (median, 32.0 mm; *p* = 0.14).

### Short-term complications

One adult with CPP succumbed from a sudden bleeding in the fourth ventricle resection cavity 3 days after surgery, resulting in a perioperative mortality of 2.7%. Transient morbidity included electrolyte imbalance (2 patients), diplopia (1 patients), facial nerve palsy (2 patients), dysphagia (2 patients), ataxia (2 patients), and epidural hematoma requiring surgical evacuation (1 patient [48]).

Permanent morbidity at last follow-up was present in 2 adult patients including diplopia (2), unilateral hypacusis (1), and dysphagia (1).

One CPC child (no. 15) developed 1 year after primary surgery a refractory focal onset secondarily generalized epilepsy, but was seizure-free after temporo-parieto-occipital disconnection surgery.

### Impairment of CSF circulation

External ventricular drainage (EVD) was placed in 6 children (35.3%) and one adult (5.3%) prior to resection due to acute intracranial hypertension. Delayed EVD placement after tumor resection was performed in 4 patients (11.1%).

A ventricular-peritoneal (5 patients) or ventricular-atrial (2 patients) shunt was placed at a median interval of 27 days (range, 0–175 days) after tumor resection. Permanent shunting was equally distributed between children and adults (17.6% vs. 21.1%; *p* = 0.80), but was more frequent in CPC (75.0%) than in aCPP (18.2%) or CPP (9.5%; *p* = 0.01).

Postoperative subdural hygroma was observed in 8 children (47.1%), occurring after 4/6 transcallosal (Fig. 2b) and after 4/9 transcortical approaches. Patients with postoperative hygroma had a significantly larger tumor (53 mm vs. 34 mm; *p* = 0.02). A subduro-peritoneal shunt was placed in 3/8 children due to progression of the hygroma, increased head circumferences (99.9 percentile), or significant cortical compression (Fig. 2c).

### Adjuvant therapy after initial surgery

Adjuvant therapy was performed in 19.4% of patients.

Four children with CPC (6 months–6.5 years) received chemotherapy. A combination of etoposide with either vincristine or ifosfamide and cisplatin or carboplatin was used. Additional radiotherapy was performed in 2/4 children with CPC, both older than 3 years. One patient with spinal metastasis at diagnosis (patient no. 15) underwent cranio-spinal irradiation (24 Gy) with boosts to the primary tumor and metastates (25.2 Gy). The older child with a small spinal metastasis and tumor cells in the CSF (patient no. 16) underwent cranio-spinal irradiation with 35.2 Gy with a local cerebral boost of 20 Gy and spinal boost of 14 Gy. None of the children with aCPP (0.2–1.2 years) received adjuvant treatment. A recurrent aCPP was totally resected after what had been assumed an initial GTR. On review, relapse was assumed to originate from a tiny residual at the Foramen of Monro.

Local radiotherapy (54 Gy) was administered to 2 adult patients with an aCPP and a highly increased MIB-Index after GTR (patient no. 34 and no. 35). One adult patient with an aCPP (no. 33) received adjuvant antiangiogenic therapy (thalidomide).

### Outcome

Four patients were lost to clinical follow-up after GTR including 3 CPP and one aCPP.

**Fig. 1:**
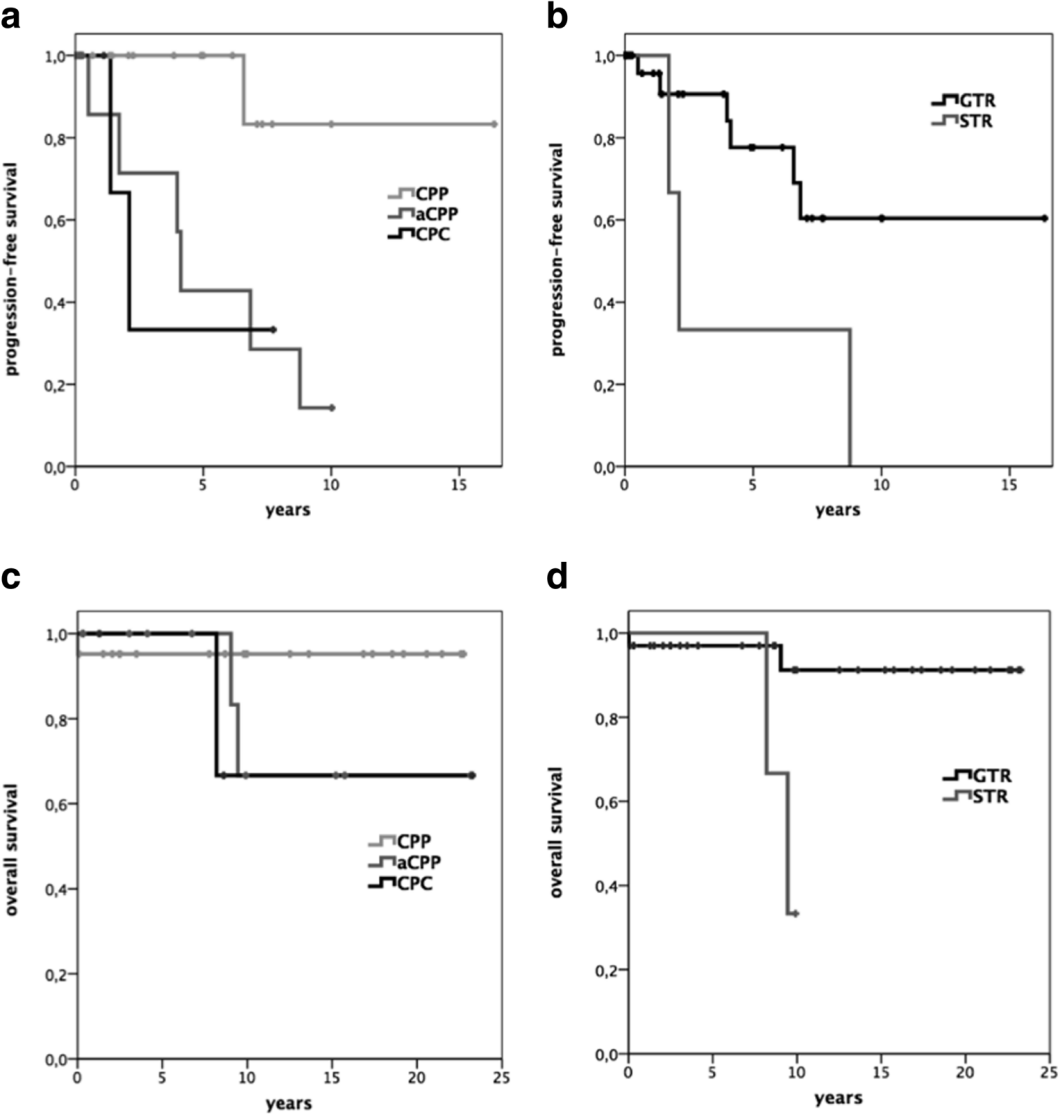
Kaplan–Meier plot displaying progression-free survival (**a**, **b**) and overall survival (**c**, **d**) in relation to histological tumor grade and extent of resection. aCPP, atypical choroid plexus papilloma; CPC, choroid plexus carcinoma; CPP, choroid plexus papilloma; GTR, gross-total resection; STR, subtotal resection

In the remaining 32 patients, median follow-up was 5.9 years (range, 0.1–20.8 years). Five-year OS for CPP was 95.2% and 100% for both aCPP and CPC. Ten-year OS for CPP was still 95.2% and 66.7% for both aCPP and CPC (Fig. 1c, d).

Extent of resection (*p* = 0.01), tumor progression (*p* = 0.04), and leptomeningeal metastasis (*p* = 0.002), but not histological grading (*p* = 0.27), were predictors for OS.

In total, four patients died (11.1%): One patient with CPP died 3 days after primary surgery due to acute rebleeding (patient no. 27). One adult died due to a secondary metastasized aCPP (patient no. 31) and one child due to a metastasized CPC (patient no. 16). One patient with an aCPP died 9 years after first surgery due to ARDS and renal failure (patient no. 36).

Five-year PFS was 100% for CPP, 85.7% for aCPP, and 33.3% for CPC (Fig. 1a, b). Histology and extent of resection were statistically significant predictors of PFS (*p* = 0.01 and *p* = 0.05 respectively).

### Tumor recurrence

Tumor recurrence was observed in nine patients (25.0%), including one CPP, six aCPP, and two CPC. Median time to relapse was 4.0 years (range, 0.5–8.8 years). Tumor progression was observed in all three patients with STR. Six of 33 (18.2%) patients with GTR had a local relapse. Tumor recurrence was observed more frequently in patients with intraoperative evident brain infiltration (77.8%) than in patients without infiltration (22.2%; *p* = 0.001) and was significantly associated with histological grading (*p* = 0.004).

**Fig. 2:**
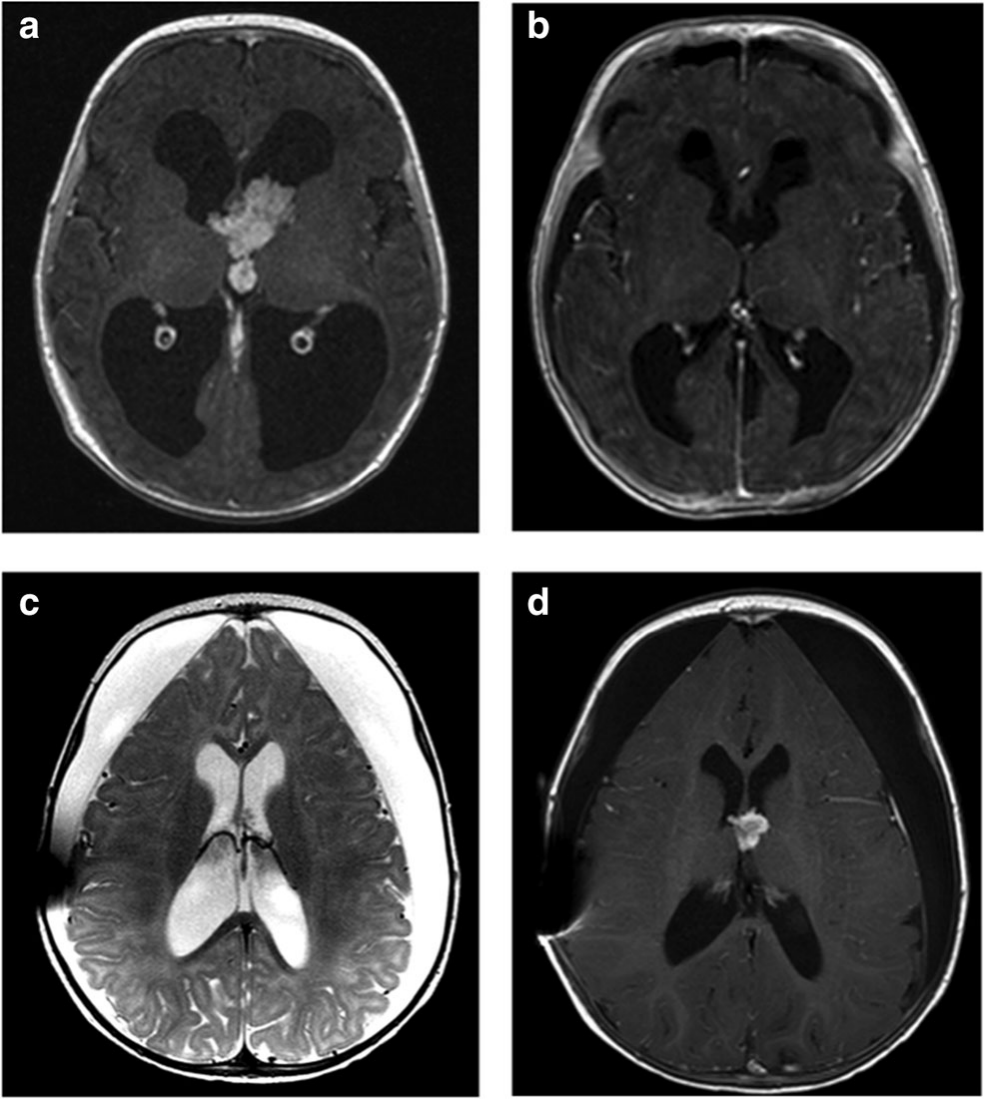
Illustrative case no. 9. A 2-month-old girl presented with increased head circumference (> 97th percentile) and a contrast-enhanced intraventricular lesion at the Foramen of Monro (**a**). Using a transcallosal approach the tumor was totally resected (**b**) and histopathology diagnosed an atypical choroid plexus papilloma. Pre-operative hydrocephalus resolved, but a subduro-peritoneal shunt was necessary due to postoperative persistent subdural hygroma (**c**). Follow-up MRI scans revealed tumor recurrence 7 months after primary surgery (**d**), which was resected via a transcallosal approach. Long-term follow-up (6.7 years) showed complete remission and excellent neurological outcome

In 6/9 cases with tumor relapse, surgical resection was performed (Fig. 2d). Time to reoperation was independent of the initial extent of resection (4.2 years for STR vs. 3.9 years for GTR; *p* = 0.79). Two patients with an aCPP required a third surgical intervention (patient no. 32 and no. 36).

One patient with a fourth ventricular aCPP (patient no. 31) underwent resection for local recurrence. This patient developed cranio-spinal dissemination and was treated with a combination of chemotherapy, gamma knife, and fractionated radiation as reported elsewhere [45].

Of the non-surgically managed relapse cases, one child with CPC (patient no. 17) was treated with intensive chemotherapy and gamma knife. In one adult with an asymptomatic recurrent CPP (patient no. 26), a wait-and-see strategy was chosen on patient’s wish, and one patient with recurrent aCPP (patient no. 34) moved abroad.

One child with a posterior third ventricle CPP underwent a second surgery years later because of a new contrast-enhancing lesion at the primary tumor site. Scar tissue only with no evidence of tumor was found at resection.

Leptomeningeal seeding was present in 8.3% of patients (one aCPP, two CPC). Two CPC cases had spinal metastases already at diagnosis (patient no. 15 and no. 16). In the aCPP patient (patient no. 31), a spinal metastasis was resected 4.6 years after primary surgery, confirming the initial histology of an aCPP.

### Differences in children and adults

**Fig. 3:**
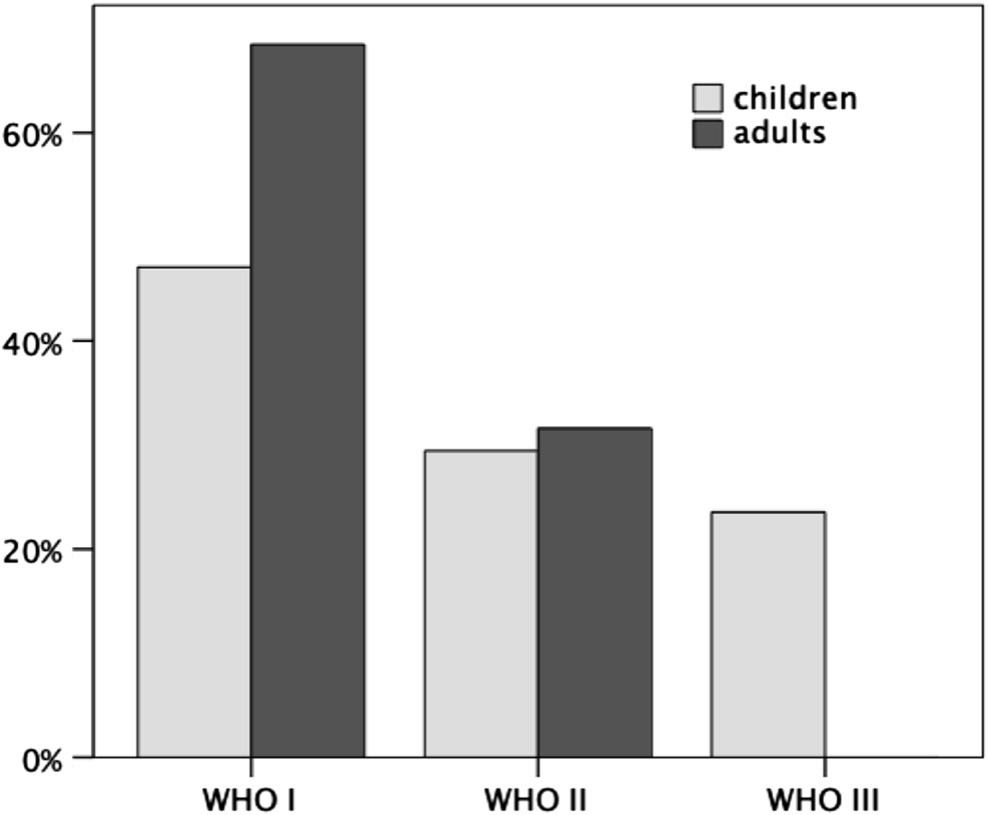
Percentage of histological grading in children and adults

Differences in symptoms at diagnosis between children and adults are shown in Table 2. Histological tumor grading was higher in children than in adults. CPP was observed more frequently in adults (68.4%) than in children (47.1%), whereas frequency of aCPP was equal in both age groups (Fig. 3). CPC were diagnosed only in children and tend to occur more frequently in male than in female patients (21.4% vs. 4.6%; *p* = 0.1).

In adults, tumors were situated exclusively infratentorially, whereas in children, tumors were located supratentorially, except for one patient, where the tumor was located within the fourth ventricle.

Intraoperatively evident parenchymal tumor infiltration and extent of resection were equally distributed between children and adults. However, blood transfusion was necessary only in children.

Overall placement of EVD was more frequent in children (41.2%) than in adults (41.2%), but did not reach statistical significance (*p* = 0.19). However, EVD placement in children was performed mostly prior to tumor resection (85.7%), whereas in adults mainly after tumor resection (75.0%; *p* = 0.04). Permanent ventricular shunting was equally distributed between children and adults (17.6% and 21.1%, respectively; *p* = 0.80), but subduro-peritoneal shunting was performed only in children (17.7%).

**Fig. 4:**
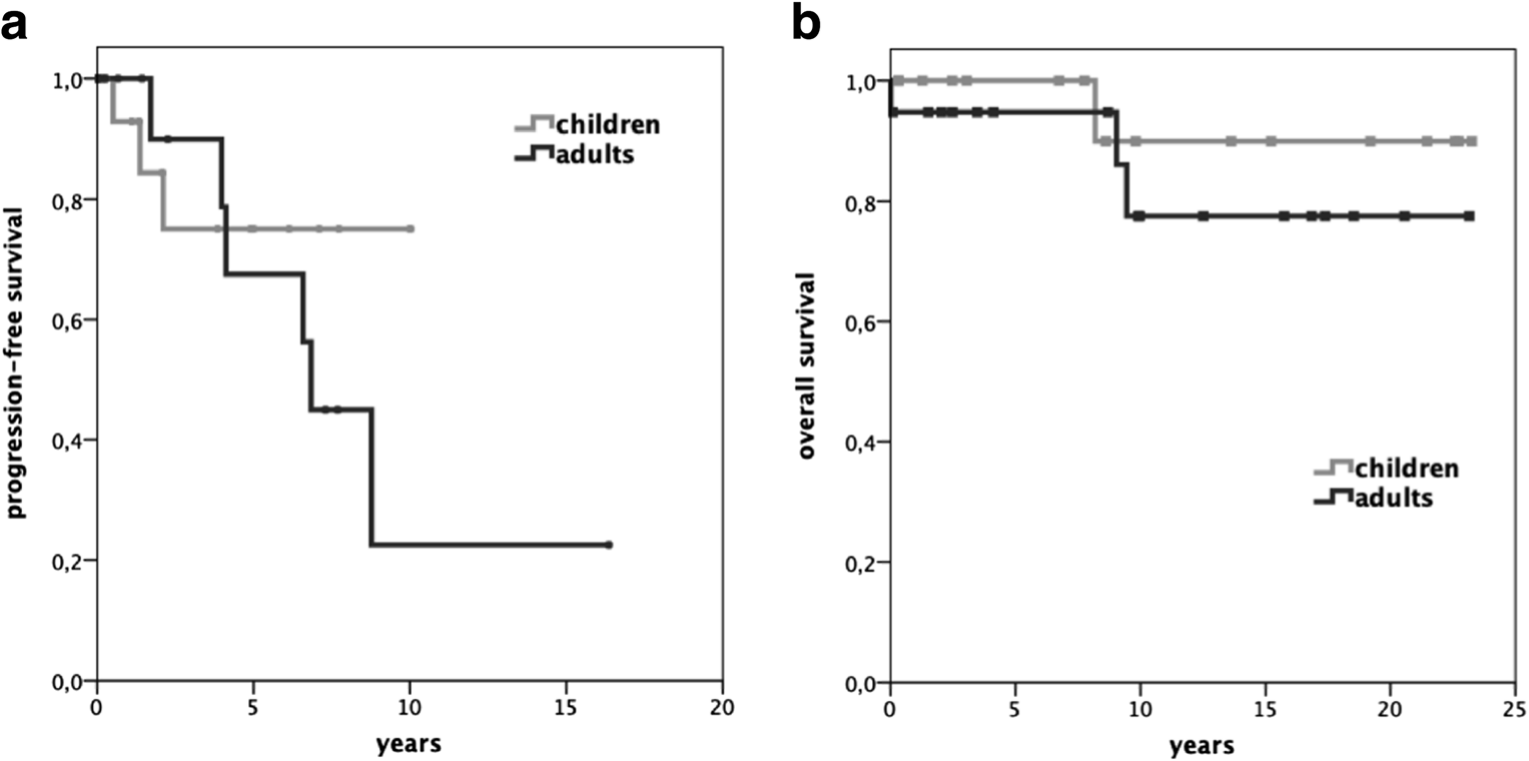
Kaplan–Meier plot displaying progression-free survival (**a**) and overall survival (**b**) for children and adults

Although, histological grading was higher in children, PFS and OS were equal in children and adults (*p* = 0.37 and *p* = 0.43, respectively; Fig. 4).

## Discussion

In our long-term cohort of a mixed pediatric and adult population, we outlined the age-specific differences for CPTs and delineated the surgically non-negligible postoperative disturbances of CSF circulation in this disease.

Although CPTs revealed higher histological grading in the pediatric population, PFS and OS were equal in children and adults. In children, the CPT was located nearly exclusively supratentorial and caused mostly symptoms of intracranial hypertension. One out of five patients—mostly children—required even an EVD ad admission. Our standardized institutional protocol is temporary CSF drainage, if required in the acute setting and reassessment of permanent shunt implantation after tumor removal. As less than half of the patients with an initial EVD required permanent drainage, the shunting rate of 19.4% was lower in our patients than reported in other series [22, 25, 30, 35]. Our rationale of initial temporary CSF drainage until tumor removal is that the hydrocephalus is mostly caused by CSF flow obstruction by the tumor. Additional potential factors in the development of hydrocephalus are CSF overproduction by the tumor itself [33] and the increase in the CSF pulse wave [6, 8, 11]. Although this tumor entity is usually given as an example of hydrocephalus related to CSF overproduction, hydrocephalus may persist after total resection. Some cases of flow obstruction can be attributed to formation of obstructive scar tissue as observed in one of our patients. However, in most cases, microbleedings or necrosis of the tumor cause impairment of CSF absorption [8, 13], which may explain the higher incidence of permanent ventricular diversion in CPC patients.

Permanent ventricular shunting was equal in both age groups, but subduro-peritoneal shunting was necessary only in children. Subdural hygroma is a common postoperative complication especially in children after transcortical and transcallosal approaches. It is caused by surgically generated ventriculo-subdural fistula on the background of the collapsed hemisphere after tumor removal [7, 8, 22, 24]. The association between tumor size and incidence of subdural hygroma may be explained by the higher incidence of hydrocephalus in larger tumors, thereby creating a cranio-cerebral disproportion with consecutive hygroma after tumor resection.

Neurological outcome can be affected significantly by perioperative morbidity [7, 42, 48] and tumor recurrence [21]. The high tumor vascularization and small circulating blood volume in infants may lead to life-threatening intraoperative bleeding with a perioperative mortality up to 12.5% [13]. Some authors suggest pre-operative embolization of feeding arteries to reduce intraoperative blood loss and improve the chance of GTR [18, 46]. However, despite high-volume experience with endovascular techniques at our center over the whole study period [4, 12, 19], we do not use embolization as the complication rate, including infarction or hemorrhage, is not negligible [18]. Therefore, precise hemostasis during resection is even more crucial to avoid fatal complications especially in children.

GTR is the most important prognostic factor in CPTs [2, 15, 39, 47]. We could show that GTR is essential for long OS and PFS, but detection of brain invasion at surgery is as well a strong predictor for tumor recurrence. The infiltrative growth of aCPP resembles the appearance of CPC, resulting in poor OS and PFS as well in aCPP cases. Patients with CPP can usually be cured by surgery alone [23, 35] and GTR in CPC patients has a significant impact on survival [5, 9, 15, 27, 34]. In our cohort, GTR was strongly associated with higher survival rates and a decreased risk of tumor recurrence. It has been reported that tumor recurrence had only an impact on survival in patients with CPC and not in patients with CPP [47]. In our cohort, tumor recurrence was in fact a negative predictor of OS, but there was no association of OS with tumor entity. We observed brain infiltration at surgery more frequently in patients with later tumor recurrence. Levy et al. also described local parenchymal invasion in some CPP patients, but without noting an impact on tumor recurrence [26]. According to our findings, we suggest to assign adult patients with intraoperative evident parenchymal invasion to a high-risk group for relapse. As tumor recurrence was observed up to 8 years after primary surgery, neuroradiological follow-up should be performed on a long-term basis.

Whereas metastatic disease in CPC is common [9, 47], dissemination of CPP or aCPP is very rare and has been reported in only a few cases so far [1, 32, 49]. We observed cranio-spinal leptomeningeal dissemination at recurrence in one adult fourth ventricular aCPP patient (9% of aCPP) with only STR at initial surgery. Therefore, MRI of the whole neuroaxis must be considered at recurrence of aCPPs and CPC. Although systemic metastases have been reported [47], we did not observe such an event in our patients.

In CPC, neoadjuvant chemotherapy has been proposed [38] but has not been used in our series. While various drug regimens are recommended after resection [2, 37], the role of radiotherapy with regard to timing, dose, and extent of the field remains to be determined [16, 29, 40]. Most series suggest a survival benefit of radiation in CPC patients [27, 47], excluding patients with germline mutation of p53 [3]. We performed adjuvant radiation of the neuroaxis in all patients with CPC older than 3 years of age, except one, who was not irradiated at primary therapy but received gamma knife treatment for a local tumor recurrence. The significantly higher 5-year OS of CPC patients (100%) than their 5-year PFS (33.3%) may be explained by the efficacy of the adjuvant therapy as reported elsewhere. [39].

Management of subtotally resected and recurrent CPPs and aCPPs remains controversial. Both of our subtotally resected aCPP of the fourth ventricle recurred. Chemotherapy is used in experimental settings only, and mostly limited to tumor recurrence, as already reported in one of our patients with a metastatic aCPP [45]. Local radiotherapy has been proposed in incompletely resected aCPPs [49]. In our series, both adults with fourth ventricular aCPP and postoperative local radiotherapy had had an initial GTR.

In most of our CPP and aCPP patients (5/7), resection was performed for local recurrence. Chemotherapy has also been reported as a viable treatment option [28, 45]. In case of metastatic recurrence, our treatment regimen was chemotherapy and/or radiation, which was preceded by resection dependent on extent of disease.

Histological grade was reported as the strongest predictor of OS [9, 47]. Five-year OS of 95.2% for CPP and 100% for both aCPP and CPC are comparatively good as observed in other series [9, 23, 27, 39, 47, 50]. One of the five children aged less than 14 months with a diagnosis of aCPP and surgery only recurred compared with a recurrence rate of 83% in our adult patients, which is in line with data of the SIOP (the International Society of Pediatric Oncology) registry [43].

aCPP can be diagnosed by histological patterns alone. However, molecular patterns beyond histological grading may account for differences in clinical outcome [31, 36, 41, 49]. It has been shown that genetic and epigenetic patterns clearly distinguish CPC from CPP and aCPP [31]. The molecular similarity of aCPP and CPP may explain the rare progression of some CPP to aCPP (< 2%) [2, 10, 21]. Methylation profiling could define risk subgroups, independent of histological grading [44]. This may explain the rare progression of grade I/II tumors to CPC [21] and is in line with data on pediatric aCPP [43]. Proliferation marker (Ki-67/MIB-1) and tumor suppressor proteins (p53) have been shown to gradually increase from CPP to aCPP and CPC with a high correlation to clinical outcome [49]. P53 mutations are found in over 50% of CPC patients [41], which are linked to increased tumor aggressiveness and decreased chemo- and radiosensitivity [14]. Not only the number of mutated copies but also structural variations of p53 are associated with worse PFS and OS [31, 41, 44]. Furthermore, age-related chromosomal alterations correlate to overall survival in CPC patients [36]. These heterogeneous molecular patterns reveal new high-risk groups beyond histological grading and may explain differences in clinical outcome. In future, the implementation of molecular data may improve therapy allocation and prediction of clinical outcome.

## Conclusion

GTR is the most important predictor of OS regardless of histological grading. The question of adjuvant therapy is still in a process of being defined, but molecular patterns will have to be included in the future to provide adequate individual treatment in a multidisciplinary approach. The intraventricular location, tumor vascularization, and impaired CSF circulation have to be considered in the surgical approach in an age-related context to reduce perioperative morbidity. Especially, the sequelae of CSF diversion must not be underestimated in the pediatric population. Long-term follow-up is necessary in all patients as metastases and relapse may occur in all subtypes.

## Abbreviations

aCPP: Atypical choroid plexus papilloma
CSF: Cerebrospinal fluid
CT: Computed tomography
CPA: Cerebellopontine angle
CPC: Choroid plexus carcinoma
CPP: Choroid plexus papilloma
CPT: Choroid plexus tumor
GTR: Gross-total resection
MRI: Magnetic resonance imaging
OS: Overall survival
PFS: Progression-free survival
STR: Subtotal resection
WHO: World Health Organization
